# The prognostic value of serum procalcitonin measurements in critically injured patients: a systematic review

**DOI:** 10.1186/s13054-019-2669-1

**Published:** 2019-12-03

**Authors:** Aziza N. AlRawahi, Fatma A. AlHinai, Christopher J. Doig, Chad G. Ball, Elijah Dixon, Zhengwen Xiao, Andrew W. Kirkpatrick

**Affiliations:** 1 Department of Surgery, University of Calgary and the Foothills Medical Centre, North Tower 10th Floor, 1403-29th St. NW, Calgary, Alberta T2N 2T9 Canada; 2 Department of Critical Care Medicine, University of Calgary, Ground Floor McCaig Tower, 3134 Hospital Drive NW, Calgary, Alberta T2N 5A1 Canada; 3 Regional Trauma Program, University of Calgary and the Foothills Medical Centre, 1403-29th St. NW, Calgary, Alberta T2N 2T9 Canada

**Keywords:** Procalcitonin, Prognosis, Trauma, Injuries, Critical care, Intensive care unit

## Abstract

**Background:**

Major trauma is associated with high incidence of septic complications and multiple organ dysfunction (MOD), which markedly influence the outcome of injured patients. Early identification of patients at risk of developing posttraumatic complications is crucial to provide early treatment and improve outcomes. We sought to evaluate the prognostic value of serum procalcitonin (PCT) levels after trauma as related to severity of injury, sepsis, organ dysfunction, and mortality.

**Methods:**

We searched PubMed, MEDLINE, EMBASE, the Cochrane Database, and references of included articles. Two investigators independently identified eligible studies and extracted data. We included original studies that assessed the prognostic value of serum PCT levels in predicting severity of injury, sepsis, organ dysfunction, and mortality among critically injured adult patients.

**Results:**

Among 2015 citations, 19 studies (17 prospective; 2 retrospective) met inclusion criteria. Methodological quality of included studies was moderate. All studies showed a strong correlation between initial PCT levels and Injury Severity Score (ISS). Twelve out of 16 studies demonstrated significant elevation of initial PCT levels in patients who later developed sepsis after trauma. PCT level appeared a strong predictor of MOD in seven out of nine studies. While two studies did not show association between PCT levels and mortality, four studies demonstrated significant elevation of PCT levels in non-survivors versus survivors. One study reported that the PCT level of ≥ 5 ng/mL was associated with significantly increased mortality (OR 3.65; 95% CI 1.03–12.9; *p* = 0.04).

**Conclusion:**

PCT appears promising as a surrogate biomarker for trauma. Initial peak PCT level may be used as an early predictor of sepsis, MOD, and mortality in trauma population.

## Introduction

Trauma is the leading cause of death during the first four decades of life and the third leading cause of death overall, across all age groups [1, 2]. Each year, trauma accounts for 41 million emergency department visits and 2.3 million hospital admissions in the USA. Of these, 192,000 die as a result of their injuries [2]. The triphasic peaks of death after injury have long been described epidemiologically. Essentially, catastrophic non-survivable injuries occur at the time of injury, with subsequent airway obstruction, respiratory failure, and especially hemorrhage predominating as the second peak. The recognition of non-recoverable head injury and especially sepsis/systemic inflammatory response syndrome-related deaths constitute the third [3, 4]. Although the global burden of traumatic death is ominous in its predicted future increase as the developing world mechanizes, great strides have recently been made in addressing both the primary peak with injury prevention and safety conscious designs, and in the second peak related to dramatic advances in resuscitation for hemorrhage, among other interventions [5, 6]. Progress in improving the outcomes of posttraumatic sepsis/SIRS is urgently required. Sepsis remains a major challenge in critically injured patients with an incidence range between 2 and 17% during the posttraumatic period, with associated mortality rates reaching as high as 23% [7].

Major trauma provokes a strong systemic inflammatory response syndrome (SIRS) early after traumatic injury as a result of tissue damage, hypotension, hypoxia, cytokine release, and inflammation [8]. The prognosis is strongly related to the posttraumatic balance between pro- and anti-inflammatory responses [9–12]. Following this induction of the inflammatory cascade is an increase in counter regulatory anti-inflammatory cytokines, which subsequently results in immunosuppression and increased susceptibility to infection and complications such as sepsis. Together, the consequence of initial injury and inflammation, subsequent immune suppression and infection, results in multiple organ dysfunction (MOD) or multiple organ failure (MOF) [9, 12]. MOD/MOF unfortunately remains the leading cause of late death following trauma [13].

Early identification of patients at risk of developing posttraumatic complications is crucial to allow the provision of early and appropriate therapy for sepsis. It has been demonstrated that prompt and appropriate management of sepsis prevents MOD, reduces mortality, and improves clinical outcomes [14, 15]. Thus, any test or clinical information that facilitates an earlier diagnosis or safely triggers the earlier appropriate treatment of sepsis may save lives. Past research has explored a number of some inflammatory markers for their prognostic value, but no clear message regarding what, if any, marker to rely on has emerged, despite promise [16–18].

This is especially true with regard to measurement of serum procalcitonin (PCT) levels, which has been of recent interest as a potential and more accurate marker of sepsis in critically ill patients. PCT is a 116-amino acid polypeptide precursor of calcitonin produced by the C cells of the thyroid gland. Healthy individuals typically have serum PCT levels less than 0.05 ng/ml. In response to bacterial endotoxins or pro-inflammatory cytokines such as interleukin-6 (IL-6) and tumor necrosis factor alpha (TNF-α), various cell types outside the thyroid gland produce PCT, resulting in up to a 1000-fold increase in levels [19, 20]. These PCT increases occur with severe inflammation, including systemic infection and especially severe sepsis [21–23]. PCT levels are thus closely related to the severity of systemic inflammation, with higher levels associated with severe sepsis, and potentially most importantly, declining levels are associated with the resolution of infection [24]. Given these unique characteristics and reliable kinetics, PCT level has emerged as a promising biomarker. Therefore, it has been suggested that serum PCT level determination may be superior to previously studied biomarkers for use in the diagnosis of sepsis, monitoring sepsis course and severity, and guiding antimicrobial therapy [16, 18, 25–27].

However, in heterogeneous populations of critically ill patients, the results concerning PCT performance remain conflicting. Therefore, in this review, we attempt to extend the scope of previous reviews by evaluating the prognostic value of serum PCT levels, in a more homogenous group of critically injured adult patients, as related to severity of injury, sepsis, organ dysfunction, and mortality.

## Materials and methods

### Search strategy

A systematic literature search was performed in accordance with the Preferred Reporting Items for Systematic Reviews and Meta-Analyses (PRISMA) Group [28] using the following databases from their inception to September 2018: PubMed, Ovid MEDLINE, EMBASE, and the Cochrane Library (see the completed PRISMA checklist in Additional file 1). Medical Subject Heading terms and keywords for procalcitonin, trauma, injury, prognosis, and predictive value were used. The search was limited to original studies on human subjects, published in the English language. Our complete search strategies are shown in Additional file 2. To identify other potentially relevant articles, the PubMed “related articles” feature was utilized and the two authors independently hand-searched the reference list of included articles and relevant reviews for additional citations.

### Study selection

Two reviewers (A.N.AR. and F.A.AH.) independently screened the titles and abstracts of all identified citations for potential eligibility. The following inclusion criteria were applied: (1) study participants (adults ≥ 16 years old trauma patients), (2) intervention (single or serial measurements of serum procalcitonin level from day of admission to trauma center or intensive care unit (ICU) following trauma), (3) comparison of prognostic performance of PCT levels compared to other potential biomarkers, (4) outcome (documentation of at least one of the outcomes of sepsis, organ dysfunction, mortality, or correlation of PCT with severity of injury), and (5) cohort or case-control study design.

Reviews, case reports, letters, conference abstracts, and editorials were excluded. Articles involving isolated injury to the central nervous system and pediatric or burn trauma were also excluded. Full-text articles of identified abstracts that were relevant with the inclusion criteria were assessed for final eligibility. The agreement between the two reviewers was assessed using Kappa statistic for the inter-rater reliability [29].

### Data extraction

Two reviewers (A.N.AR. and F.A.AH) independently extracted data using a standardized recording tool to record the study design and setting, year of publication, country of origin, number of study participants, participant clinical characteristics, PCT testing system, kinetics of PCT, markers other than PCT, and study outcomes.

We defined the terms “SIRS” and “Sepsis” according to the American College of Chest Physician/Society for Critical Care Medicine [30], which supported definition of SIRS, sepsis, and severe sepsis as most studies were published prior to the release of Sepsis-3 [31].

### Methodological quality assessment

The risk of bias of the included studies was assessed by two independent investigators (A.N.AR. and F.A.AH) using the Quality in Prognosis Studies (QUIPS) tool developed by Hayden et al. [32]. This tool consists of 30 criteria divided into six domains: patient selection, study attrition, prognostic factor measurement, outcome measurement, confounding measurement and account, and statistical analysis and reporting. Each criterion was scored using “yes,” “no,” or “unclear.” This scoring led to the overall judgment of “low,” “moderate,” or “high” risk of bias per domain. We considered a study to be of high quality when the bias was rated as low or moderate with respect to almost all of the domains. Conversely, a study was considered to be of low quality when the bias was rated high in most of the bias domains. Disagreements were resolved by consensus.

## Results

### Literature search

**Fig. 1:**
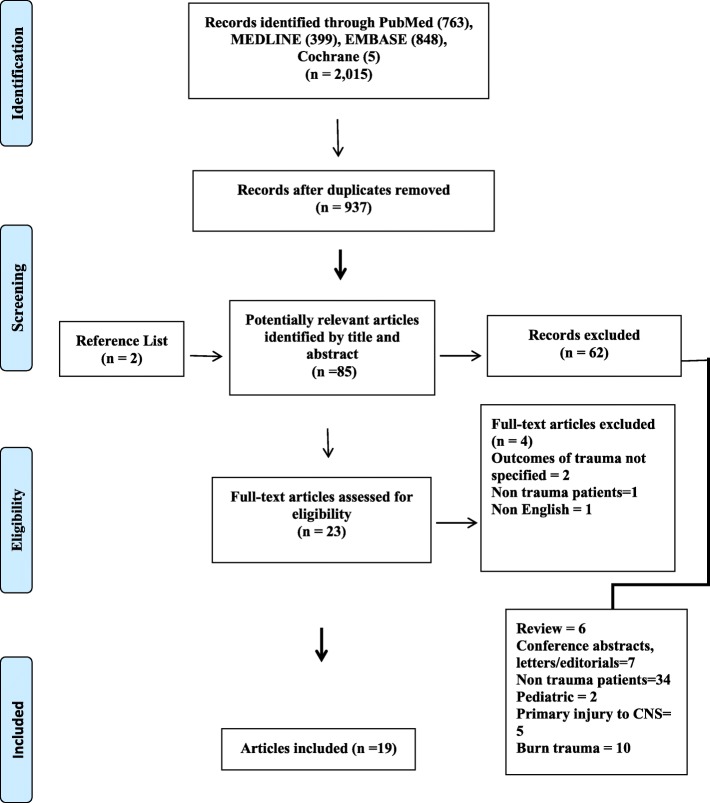
Flow diagram of selected studies for review

Figure 1 presents a flow diagram of study identification and subsequent inclusion. Among 2015 citations identified by the search, 19 studies fit the inclusion criteria including 4146 patients with multiple trauma. There was an excellent inter-investigator agreement on the inclusion of full-text articles in the systematic review (*κ* statistic = 0.88; 95% CI = 0.65–1.00).

Due to heterogeneity between studies with respect to definition of clinical outcomes and statistical methods, it was not possible to statistically pool the results. Instead, findings were reported descriptively.

### Characteristics of the included studies

Table 1 details the characteristics of the included studies. The included studies were published from 1998 to 2016, while the majority were published after 2006 (13/19; 68.4%). Sixteen studies were conducted in Europe [34, 37–51] (84.2%), two were conducted in Asia [33, 35] (10.5%), and one was conducted in the USA [36] (5.2%). All studies were observational and non-interventional. Eleven studies were prospective cohort [34, 36–39, 41–43, 45, 46, 51], six studies were prospective case-control [33, 35, 44, 47–49], one study was retrospective cohort study [40], and one study was retrospective case-control [50].

**Table 1:** Details of study characteristics of included studies

Study	Study design	Study setting	No. of patients	Mortality %	Peak PCT level	PCT level predicting outcomes		
						Sepsis	MODS	Death
Ren et al. [33] (China, 2016)	Prospective case-control	Trauma surgical department	56	–	Days 1 and 2	Yes	–	–
Wojtaszek et al. [34] (Poland, 2014)	Prospective cohort	–	45	Data not shown	Day 1	No	–	Yes
Rajkumari et al. [35] (India, 2013)	Prospective case-control	SICU	275	10	–	Yes	No	–
Sakran et al. [36] (USA, 2012)	Prospective cohort	Trauma ICU	102	13	Days 1 and 2	Yes	–	Yes
Haasper et al. [37] (Germany, 2010)	Prospective cohort	ICU	94	12	Days 2 and 3	No	Yes	–
Keel et al. [38] (Switzerland, 2009)	Prospective cohort	Trauma center	83	12	Day 1	Yes	–	–
Castelli et al. [39] (Italy, 2009)	Prospective cohort	ICU	94	5	Day 1	Yes	Yes	–
Billeter et al. [40] (Switzerland, 2009)	Retrospective cohort	SICU	1032	10	Day 1	Yes	–	–
Maier et al. [41] (Germany, 2009)	Prospective cohort	–	74	–	Day 1	–	–	–
Balci et al. [42] (Turkey, 2009)	Prospective cohort	SICU	113	44	Days 1 and 7	Yes	–	Yes
Castelli et al. [43] (Italy, 2006)	Prospective cohort	ICU	49	–	Day 1	Yes	Yes	–
Ertugrul et al. [44] (Turkey, 2006)	Prospective case-control	SICU	41	–	–	No	–	–
Meisner et al. [45] (Germany, 2006)	Prospective cohort	ICU	90	17	Day 1	Yes	No	Yes
Egger et al. [46] (Austria, 2004)	Prospective cohort	ICU	26	–	–	Yes	–	–
Hensler et al. [47] (Germany, 2003)	Prospective case-control	Trauma center	137	11	Days 1 + 2 combined	No	Yes	No
Andermahr et al. [48] (Germany, 2002)	Prospective case-control	ICU	133	21	Day 1	No	–	–
Oberholzer et al. [49] (Switzerland, 2000)	Prospective case-control	Trauma center	1276	7	Day 1	Yes	Yes	–
Wanner et al. [50] (Germany, 2000)	Retrospective case-control	ICU	405	23	Days 1 and 3	Yes	Yes	No
Mimoz et al. [51] (France, 1998)	Prospective cohort	SICU	21	14	Day 1	No	Yes	–

*Abbreviations*: *ICU* intensive care unit, *SICU* surgical intensive care unit, *PCT* procalcitonin, *MODS* multiple organ dysfunction syndrome

**Fig. 2:**
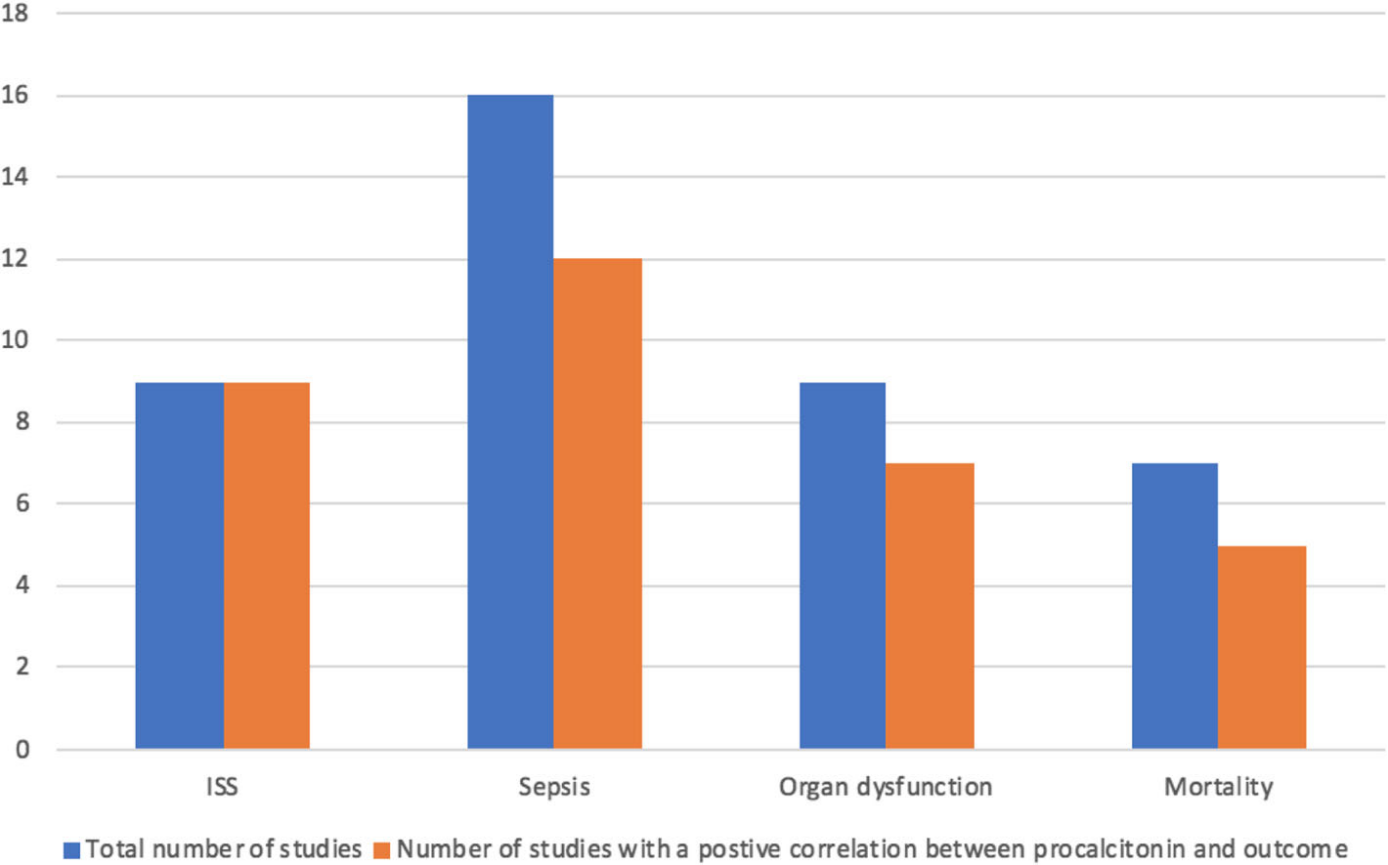
Studies evaluated the role of procalcitonin levels in predicting clinical outcomes following trauma

All studies evaluated the role of PCT in predicting at least one clinical outcome in critically injured patients (Fig. 2). Twelve studies (63.3%) were conducted in the critical care units [35–37, 39, 40, 42–45, 48, 50, 51]. Overall, the aggregate study population included a total of 4146 patients with trauma in whom serum PCT levels were used to predict posttraumatic complications. The mean age ranged between 34 and 49 years. The mean injury severity score ranged from 21 to 32. The mechanism of injury was blunt and/or penetrating trauma. All studies were civilian, with none comprising military populations or combat injuries.

### Risk of bias assessment

**Fig. 3:**
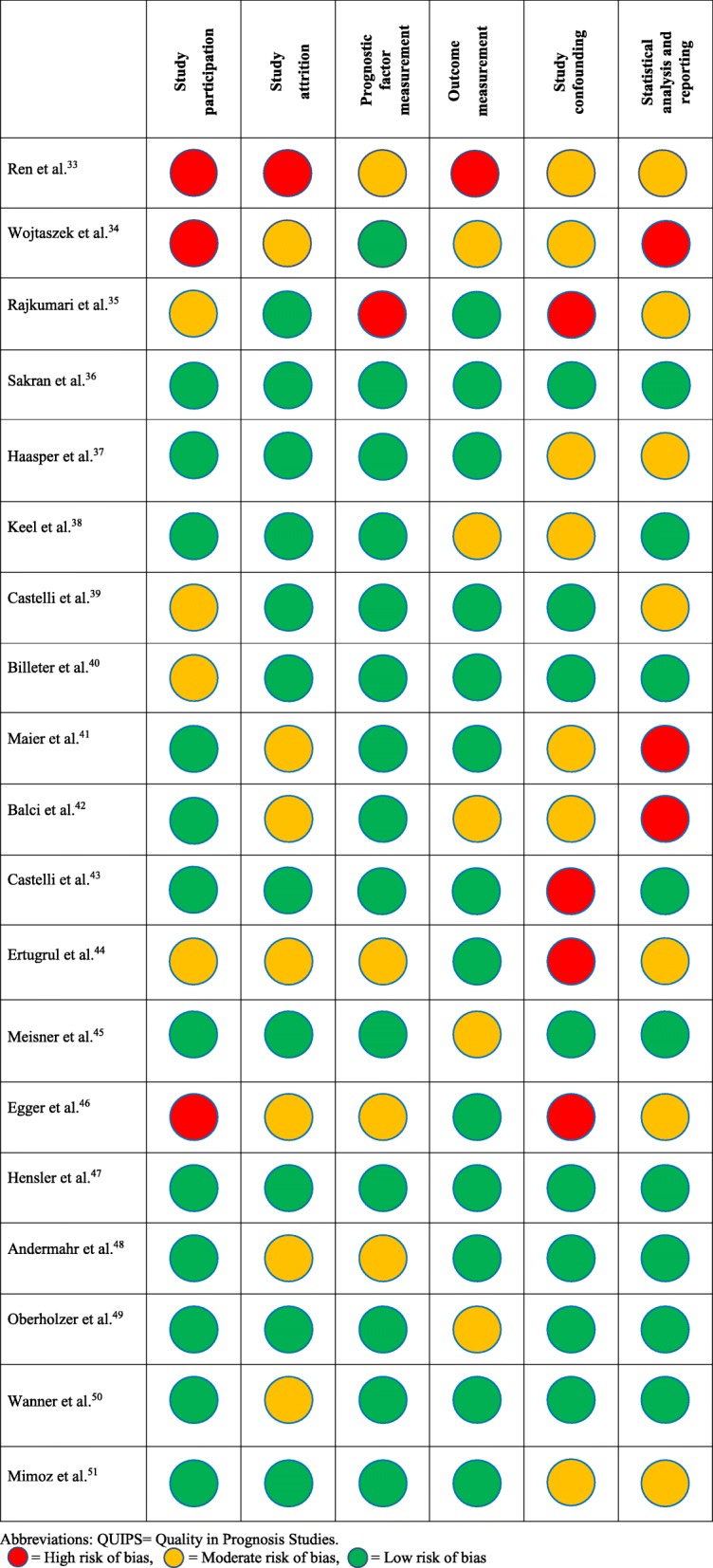
Assessment of risk of bias using QUIPS tool

Figure 3 summarizes the risk of bias assessment for all included studies. Most studies were assessed to be of low to moderate risk of bias. Nine studies demonstrated high risk of bias in at least one domain [33–35, 37, 39, 41, 42, 44, 46]. Study confounding domain was deemed to be at moderate to high risk of bias in 14 studies (73.7%) [33–35, 37–46, 51]. The majority did not account for potential confounding factors in the study designs and/or made adjustment for the effects of the confounders in the analysis, while 21.1% of these studies did not name any confounder [35, 43, 44, 46]. The risk of bias was moderate to high in the domain of statistical analysis in 10 studies (52.6%) [33–35, 37, 39, 41, 42, 44, 46, 51]. Only 7 studies used statistical models to assess prognostic relationships [36, 37, 40–42, 45, 47]. In one study, mortality data were presented in graph only [34].

### Kinetics of PCT

PCT levels were measured from the serum sample in all studies, using immunoluminometric assay (LUMItest) in 11 studies (57.8%) [38, 40, 42–45, 47–51] and Kryptor Assay in 3 studies (15.7%) [36, 37, 41]. Other techniques used to determine PCT levels were Roche Cobas e 411 [33], VIDAS system [35], enzyme-linked fluorescent immunoassay (ELFA) [34], and chemiluminescence analyzer [39]. Most studies showed rapid kinetics of PCT levels with peak levels reached on day 1 post trauma [33, 36, 38–43, 45, 47–51], and to a lesser extent on day 2 [34, 37]. PCT levels declined rapidly thereafter towards the normal range. Sakran et al. [36] and Haasper et al. [37] demonstrated that a biphasic rise in PCT after day 7 was associated with development of sepsis.

### Correlation between PCT levels and injury severity and injury pattern

Nine studies (47.4%) [33, 39, 41, 42, 45–47, 50, 51] assessed the correlation between initial PCT level and the severity of injury using Injury Severity Score (ISS) [52]. All studies showed a correlation between initial PCT levels and ISS. When patients were categorized into those with severe trauma (ISS > 20) or moderate trauma (ISS < 20), the initial PCT was significantly higher in patients with severe trauma [41, 42, 45, 50].

Four studies assessed the association between PCT level and injury pattern, three of which showed that serum levels of PCT were higher among patients with abdominal injury [35, 41, 45], whereas one study showed no correlation between PCT level and injury pattern [50].

### The value of serum PCT levels in differentiating sepsis from non-infectious systemic inflammation in injured patients

Sixteen studies (84.2%) assessed the utility of serum PCT level as a marker for sepsis [33, 35–40, 42–50]. In patients who developed systemic or septic complications, the kinetic of PCT was similar to those without complications. After reaching the peak level on day 1 after trauma, an immediate decline was observed towards normal range [36, 38, 40, 45, 50]. However, the initial peak PCT was significantly higher in patients who subsequently developed sepsis compared to those without sepsis and the difference remained significant between the two groups during the study period [33, 36, 38–41, 45, 46, 49, 50]. Further, patients who developed sepsis demonstrated a significant increase of peak PCT levels compared with patients with non-infectious systemic inflammation [36, 42, 43, 50]. Because the severity of injury itself can significantly influence circulating PCT levels, Wanner et al. demonstrated a 3.9-fold increase of initial peak PCT levels in injured patients with sepsis compared with patients with SIRS [50]. In addition, in a subgroup analysis of injured patients with low ISS (ISS < 25) compared with patients with high ISS (ISS ≥ 25), PCT levels remained significantly elevated in patients with sepsis compared with patients without sepsis in those subgroups [50]. Castelli et al. observed a significant increase in PCT level in trauma patients at day of sepsis diagnosis as compared with levels measured on day 1 before the diagnosis [39]. Rajkumari et al. reported that all patients with serum PCT level > 2 ng/ml developed infections after trauma [35]. Three studies measured the sensitivity and specificity for the diagnosis of sepsis as compared with non-infectious systemic inflammation. With cutoff point of procalcitonin level of 1.5 ng/mL, the sensitivity to detect sepsis ranged from 42 to 76%. In those same studies, the specificity ranged from 73 to 77% [45, 47, 50].

The most common identified sources of infection resulting in sepsis were the lungs [36, 39, 40, 42, 43, 45, 48, 50], bloodstream [36, 39, 40, 42, 43, 45], urinary tract infection [36, 39, 40, 45, 48], soft tissue infection [39, 42, 43, 45, 48, 50], peritonitis [39, 43, 45, 50], and bacterial meningitis [43, 50]. However, four studies reported no significant difference in the PCT level between patients who developed sepsis and those who did not [37, 44, 47, 48], although one study evaluated the predictive value of PCT level for the development of posttraumatic pneumonia and reported higher PCT levels among patients who developed pneumonia compared with those without, but the difference did not reach significance [44].

### The value of serum PCT levels in predicting the future occurrence of MODS in injured patients

Nine studies (47.4%) assessed the utility of serum PCT level in predicting the future occurrence of MOD and/or MOF in injured patients [35, 37, 39, 43, 45, 47, 49–51]. Studies used either the Sequential Organ Failure Assessment (SOFA) [53] or the Multiple Organ Failure Score developed by Goris to assess the severity of organ dysfunction [54, 55]. Seven studies demonstrated significantly higher initial PCT levels in patients who subsequently developed MODS compared to those patients without MODS [37, 39, 43, 47, 49–51]. Haasper et al. observed an elevated PCT level preceding the development of MODS by 3 days [37]. Hensler et al. divided patients with MOF into early and late MOF using day 3 as a cutoff point to avoid biased results by patients who developed MOF already on day 1. Results remained significant for both groups when compared with patients without MOF [47]. However, two studies showed no correlation between PCT levels and severity of organ dysfunction [35, 45].

### The value of serum PCT levels in predicting mortality in injured patients

Among seven studies assessing the value of procalcitonin serum level determination in predicting death following trauma, three studies demonstrated significant elevation of PCT levels in non-survivors compared with that in survivors [34, 42, 45]. The difference in PCT level between the two groups remained significant during the first week after trauma in two of those studies [42, 45]. Meisner et al. showed that by the end of the first week, the difference between survivors and non-survivors increased up to 15-fold in patients with fatal outcome [45]. A study by Sakran et al. demonstrated significantly increased mortality among patients with a PCT level of ≥ 5 ng/ml compared with that of patients with less than 5 ng/ml, with odds ratio of 3.65 (95% CI 1.23–4.61, *p* = 0.01) [36]. Conversely, two studies reported no association between PCT level and fatal outcome [47, 50].

### Prognostic value performance of PCT levels compared to other potential biomarkers

Fourteen studies (73.7%) assessed serum PCT levels and other biomarkers simultaneously to predict posttraumatic complications (Table 2). Five studies demonstrated slow induction of C-reactive protein (CRP) after trauma with peak levels reaching on day 3 after trauma [38–40, 43, 45]. PCT was superior to CRP in predicting septic complications in these studies.

**Table 2:** Prognostic values of other biomarkers studied simultaneously with serum PCT levels

Study	Other biomarkers	Comments on other biomarkers
Ren et al. [33]	HSP70, WBC	- HSP70 and WBC levels were elevated at 1–6 h post injury while PCT increased 24 h post - Magnitude of HSP70 increased was related to the severity of injury - Increased HSP70 24 h post injury suggested infection
Rajkumari et al. [35]	CRP	- No difference in CRP levels between patients with and without sepsis - PCT and CRP did not correlate with SOFA score
Haasper et al. [37]	IL-6	- IL-6 levels peaked on day 0, while PCT peaked levels peaked on day 1 - Significant difference in IL-6 and PCT levels between patients with and without MODS - No difference in IL-6 and PCT levels between patients with and without sepsis
Keel et al. [38]	PSP, CRP, IL-6	- Significant difference in PSP levels between patients with and without sepsis during hospital stay - Slow induction of CRP with peak levels reaching day 3. Significant difference in CRP levels between patients with and without sepsis on day 7 after trauma - Peak IL-6 levels of day 0 after trauma. Significantly higher IL-6 levels in septic patients after day 5 compared to patients with no infection - Peak PCT on day 1. Significant PCT levels between patients with sepsis and without on days 1, 3, 5, 7, and 14 - No difference in CRP, PCT, and IL-6 levels between patients with sepsis and local infection
Castelli et al. [39]	CRP	- No difference in CRP levels between patients with and without sepsis - CRP did not correlate with SOFA score
Billeter et al. [40]	CRP, IL-6, lactate	-IL-6 peaked on day 1 after trauma - Significant difference in IL-6 levels between patients with or without sepsis on days 3 and 5. No difference after day 5 - Slow induction of CRP with peak levels reaching between days 3 and 7 - Significant difference in CRP levels between patients with or without sepsis on days 5, 7, and 14 - Insufficient 24-h lactate clearance was associated with high rate of mortality and sepsis
Balci et al. [42]	CRP	- CRP levels were higher only in cases of severe sepsis or septic shock, but not in cases of sepsis alone - Significant difference in CRP level between survivors and non-survivors on days 1 and 7
Castelli et al. [43]	CRP	- Slow induction of CRP after trauma - No correlation between CRP levels and sepsis - CRP levels correlated with SOFA score
Ertugrul et al. [44]	CRP	- No difference in CRP levels between infected and non-infected groups
Meisner et al. [45]	CRP	- CRP levels peaked on day 3 (slow induction) - No correlation between CRP levels and posttraumatic complications including sepsis, MODS, and mortality
Egger et al. [46]	PMN migration, CRP, IL-6, IL-8, NT, lactate, cortisol, Elastase, MDA	- PMN migration was a highly sensitive predictive marker for infection - No difference in the other biomarker levels between infected and non-infected groups
Hensler et al. [47]	Neopetrin (NT)	- NT level decreased on day 0 after trauma, followed by an increase on days 1 and 2 - Both PCT and NT were unable to differentiate between patients who developed sepsis or not - No difference between PCT or NT levels of survivors and non-survivors - No difference in NT levels between patients with and without MOF
Oberholzer et al. [49]	IL-6	Both PCT and IL-6 levels peaked on day 1 after trauma Both PCT and IL-6 levels were significantly higher in septic patients compared with patients without sepsis Both PCT and IL-6 levels were significantly higher in patients who developed MODS compared with patients without MODS
Mimoz et al. [51]	CRP	- PCT levels peaked on day 1 while CRP levels peaked on day 2 after trauma - Both peak PCT and CRP levels were higher in patients who subsequently developed MODS

*Abbreviations*: *HAI* hospital-acquired infection, *CRP* C-reactive protein, *IL* interleukin, *PCT* procalcitonin, *NT* neopetrin, *PMN* polymorphonuclear leucocyte, *PSP* pancreatic stone protein, *SOFA* sequential organ failure assessment, *MODS* multiple organ dysfunction syndrome, *MDA* malondialdehyde

Interleukin 6 (IL-6) has similar kinetics to those of PCT. IL-6 increases during the early phase after trauma with peak levels reached on day 0 after trauma [37, 38, 40, 49]. While Billeter et al. observed a significant difference in IL-6 levels between patients who developed sepsis and those who did not on days 3 and 5 after trauma [40], Keel et al. observed this difference only after day 5 [38].

Haasper et al. demonstrated low sensitivity of IL-6 for predicting sepsis and low specificity for predicting both sepsis and MODS [37].

## Discussion

Serious traumatic injuries often induce an early overwhelming systemic inflammation and a late immune paralysis that can disrupt immune system homeostasis and predispose patients to septic complications with an ultimately fatal outcome. Ongoing international efforts have produced substantial progress into understanding how trauma affects the immune system, although the overall picture is confusing and still in its relative infancy. Nonetheless, irrespective of the exact molecular mechanisms involved, it remains true that the basic principle of initiating appropriate antibiotics early while searching for and correcting the source of septic complications will save lives [56]. However, the indiscriminate use of broad-spectrum antibiotics based on the mere chance that a patient may be developing sepsis cannot be justified, and such practices have grave implications for all of the critically ill/injured in the future. Thus, a biomarker that may help identify patients who are either developing or at high risk for developing sepsis before this becomes otherwise clinically apparent might ameliorate such adverse outcomes following trauma.

Since its first description in 1993 by Assicot et al., authors have described a strong and generally consistent association between serum PCT level and the subsequent clinical course of severely traumatized patients [24]. Early elevation of PCT is related to the severity of trauma and magnitude of tissue injury. Trauma patients with SIRS have initially elevated levels of PCT. However, PCT levels are only mildly elevated in non-infectious SIRS. Significantly elevated PCT levels correlate with a substantially increased risk for septic complications. Observational studies have shown that most patients with non-infectious SIRS have an inflammatory-mediated procalcitonin level ranges from 0.3 to 0.8 ng/mL [57–60], while studies on septic patients (from any source) in an ICU setting have shown PCT levels range from 4.5 to 12.0 ng/mL [61–64]. In addition, rapid decline to normal levels most often indicates resolution of systemic inflammation/infection. Therefore, under these circumstances, the initial peak PCT level can reliably differentiate between infectious and non-infectious SIRS in critically injured patients and hence outperform other biomarkers such as C-reactive protein and IL-6 [65, 66]. Since the increase in PCT level usually precedes the onset of clinical symptoms, it allows earlier detection of infection than the conventional standard methods.

In our review, four studies did not demonstrate an association between PCT level and development of sepsis. The variability in the results might be related to the lack of consistency in defining sepsis and lack of consensus gold standard for defining infection per se. This could have misclassified patients as having systemic inflammation if potentially infected patients did not exhibit clinical signs or in whom bacterial cultures were negative. In the study of Ertugul et al., a single time point PTC was used to predict the development of hospital-acquired infection (HAI) [44]. However, given the complexity of the immune response after trauma which is influenced by both patient-specific factors and injury-specific factors, one single parameter may not be able to adequately predict the clinical course. Andermahr et al. reported no significant difference in PCT levels between patients with or without pneumonia [48]. However, not every patient with an infection is septic. Studies have shown that PCT levels are significantly higher in patients with sepsis than those with an isolated pulmonary infection [67].

Sepsis and MOF are the predominant cause of late death in trauma [13]. A review by Ciriello et al. reported the usefulness of PCT level in predicting sepsis course in trauma population, thus allowing early diagnosis of MODS [18]. Our review suggests there is also utility of PCT level in predicting mortality. In this group of patients, early recognition of septic complications through the use of a specific and rapid marker for infection and hence early therapeutic decision may have an impact on survival and improve outcomes. In our reviews, most studies demonstrated a significant increase in early PCT level in non-survivors compared with survivors after trauma. Two studies did not show an association between PCT level and late mortality. In the study of Wanner et al., the incidence of sepsis was low (11.1%) and 70% of the deaths occurred less than 72 h after trauma [50]. In the study of Hensler et al., a small number of patients died after trauma [47]. Furthermore, the time frames in the definition of mortality varied across studies; some studies used ICU mortality, whereas other studies used 28-day mortality. These limitations could probably preclude achieving a substantial difference. Nevertheless, sepsis is clearly associated with high mortality. Studies have found that the mortality from trauma-related sepsis is significantly higher than mortality from trauma alone [68, 69]. PCT has been shown to reflect the prognosis of sepsis in septic patients. Svoboda et al. observed a tendency to reduce mortality rate in septic patients after multiple trauma or major surgery using PCT in guiding early re-intervention [70].

To our knowledge, this is the first systematic review to comprehensively assess the prognostic value of PCT level in relation to four outcomes including severity of injury, development of sepsis and MODS, and mortality in trauma patients. Most studies in our review were prospective, which allowed the study of the kinetics of PCT level from day 0 post trauma, and its association with the occurrence of complications later in the subsequent clinical course, hence accurately assessing the prognostic value of PCT level.

This review has several limitations. We did not include non-English publications in our review. Studies conducted in mixed Medical-Surgical ICU that included trauma cohort were excluded due to combined data. The quality of the primary studies varied with main issue related to confounding variables. Statistical methods used in the outcome assessment varied across studies, which made combining results in a meta-analysis difficult, which certainly represents a drawback of the current review. There was lack of a consensus definition of the term, severe trauma. This could induce a high heterogeneity among trauma patients due to variations in the immunological responses depending on the injury pattern.

## Conclusions

Despite the limitations identified during this review, PCT seems to hold promise as a surrogate biomarker for trauma. Initial peak PCT level may be used as an early predictor of severity of injury, development of sepsis and MOD, and mortality in trauma population. Serum PCT levels may contribute to the identification of patients who may benefit most from more aggressive management. However, further studies, preferably prospective randomized controlled multicenter open-label intervention trials, are necessary to investigate the impact of procalcitonin-guided decision-making on the clinical outcomes in the trauma setting.

## Supplementary information

**Additional file 1.** Preferred Reporting Item for Systematic Reviews and Meta-Analysis (PRISMA) checklist.

**Additional file 2.** Search strategies.

## Data Availability

ANAR and FAAH had full access to all data in the study and take responsibility for the integrity of the data and the accuracy of the data analyses.

## Abbreviations

APATCHE: Acute Physiology And Chronic Health Evaluation
CRP: C-reactive protein
ELFA: Enzyme-linked fluorescent immunoassay
HAI: Hospital-acquired infection
IAH: Intra-abdominal hypertension
ICU: Intensive care unit
IL: Interleukin
ISS: Injury Severity Score
MDA: Malondialdehyde
MOD: Multiple organ dysfunction
MODS: Multiple organ dysfunction syndrome
MOF: Multiple organ failure
NT: Neopetrin
OR: Odds ratio
PCT: Procalcitonin
PMN: Polymorphonuclear leucocyte
PRISMA: Preferred Reporting Item for Systematic Reviews and Meta-Analysis
PSP: Pancreatic stone protein
QUIPS: Quality in Prognosis Studies
SIRS: Systemic inflammatory response syndrome
SOFA: Sequential Organ Failure Assessment
TNF-α: Tumor necrosis factor alpha
