# Supplementary material for: The prognostic value of serum procalcitonin measurements in critically injured patients: a systematic review
Source: Crit Care. 2019 Dec 3;23:390. doi: 10.1186/s13054-019-2669-1 (PMC6892215; doi:10.1186/s13054-019-2669-1)
Supplement: Supplementary file 2 — Additional file 2. Search strategies. [file 13054_2019_2669_MOESM2_ESM.docx]

**Search Strategy**

**PubMed**

(("procalcitonin"[Supplementary Concept] OR "procalcitonin"[All Fields]) OR PCT[All Fields]) AND (("injuries"[Subheading] OR "injuries"[All Fields] OR "trauma"[All Fields] OR "wounds and injuries"[MeSH Terms] OR ("wounds"[All Fields] AND "injuries"[All Fields]) OR "wounds and injuries"[All Fields]) OR ("wounds and injuries"[MeSH Terms] OR ("wounds"[All Fields] AND "injuries"[All Fields]) OR "wounds and injuries"[All Fields] OR "injury"[All Fields]) OR ("injuries"[Subheading] OR "injuries"[All Fields] OR "wounds and injuries"[MeSH Terms] OR ("wounds"[All Fields] AND "injuries"[All Fields]) OR "wounds and injuries"[All Fields]) OR ("wounds and injuries"[MeSH Terms] OR ("wounds"[All Fields] AND "injuries"[All Fields]) OR "wounds and injuries"[All Fields] OR "wound"[All Fields]))

**N=763**

**Medline**

1. exp "Wounds and Injuries"/

2. (trauma or injur* or wound*).kw,tw.

3. 1 or 2

4. procalcitonin.mp.

5. 3 and 4

N= 399

**EMBASE**

1. injury/ or abdominal injury/ or blunt trauma/ or crush trauma/ or multiple trauma/ or pelvis injury/ or seatbelt injury/ or wound/

2. (trauma or injur* or wound*).kw,tw.

3. 1 or 2

4. exp procalcitonin/

5. 3 and 4

N=848
